# Nocebo Effects on Perceived Muscle Soreness and Exercise Performance Following Unaccustomed Resistance Exercise: A Pilot Study

**DOI:** 10.3390/jfmk5020040

**Published:** 2020-06-09

**Authors:** Blake H. McLemore, Sarah G. McLemore, Rebecca R. Rogers, Joseph A. Pederson, Tyler D. Williams, Mallory R. Marshall, Christopher G. Ballmann

**Affiliations:** Department of Kinesiology, Samford University, 800 Lakeshore Dr., Birmingham, AL 35229, USA; bmclemor@samford.edu (B.H.M.); sfortin@samford.edu (S.G.M.); rrogers1@samford.edu (R.R.R.); jpederso@samford.edu (J.A.P.); twilli11@samford.edu (T.D.W.); mmarshal@samford.edu (M.R.M.)

**Keywords:** range of motion, rate of perceived exertion eccentric, negative-belief

## Abstract

The purpose of this study was to investigate the effects of nocebo administration on perceived soreness and exercise performance following unaccustomed resistance exercise. Untrained males were randomly assigned to one of two treatments: (1) control or (2) negative-belief. For the negative-belief group, participants were given a capsule before exercise containing 400 mg of an inert substance (gluten-free cornstarch) and were told the supplement would increase muscle soreness. The control group received no treatment. An algometer and pain scale was used to obtain soreness, and a goniometer was used to measure elbow range of motion (ROM). Participants completed an eccentric bicep curl pyramid with their non-dominant arm. Rate of perceived exertion (RPE) and repetitions were recorded. Then, 48 h after the initial exercise bout, participants repeated all procedures. Perceived soreness, ROM, average RPE, and total repetitions performed were analyzed. Perceived soreness was significantly higher in both control and negative-belief groups 48 h after exercise (*p* < 0.001; η^2^ = 0.23). ROM was significantly lower 48 h post in the negative-belief group (*p* = 0.004; d = 1.83) while no differences existed for controls (*p* = 0.999; d = 0.16). Average RPE was unaffected between groups (*p* = 0.282; η^2^ = 0.07). Total repetitions were significantly lower 48 h post in the negative-belief group (*p* < 0.001; d = 2.51) while no differences existed for the controls (*p* = 0.999; d = 0.08). Findings suggest that 48 h after unaccustomed resistance exercise, negative expectation does not worsen soreness but hinders ROM and exercise performance.

## 1. Introduction

High-intensity and unaccustomed exercise can often be associated with negative side effects, many of which may be exacerbated by psychological influences [1]. The nocebo effect is a phenomenon in which an expectation of a detrimental outcome leads to exacerbations of negative symptoms [2]. Decreases in pain tolerance [3], increased anxiety [4], alteration of the rate of perceived exertion [5], and impaired aspects of physical performance [6,7] have all been associated. However, the influence of the nocebo effect on exercise and muscle soreness is currently unclear.

The nocebo effect has been widely studied in the context of pain and pain tolerance, with many investigations reporting hyperalgesia [2,8,9,10]. However, much less is known about the nocebo effect in sport and exercise. Beltranena et al. found that informing participants that fasting 24 h prior to exercise would decrease performance resulting in decreased cycling performance but administration of a calorie-free beverage improved performance to fed-state levels [11]. Interestingly, Hurst et al. showed that nocebo treatment worsened repeated sprint time, but the effect was moderated on the participant’s intension of supplement use [12]. Bottoms et al. showed that while nocebo treatment increased local ratings of perceived exertion (RPE), there were no changes in peak minute power during arm crank exercise compared to controls [5]. Furthermore, Cheung et al. reported that despite negative expectations and nocebo treatment, ischemic precondition prior to exercise was able to improve exercise performance [13]. Few studies to date have investigated nocebo effects on resistance exercise performance. Given disparities between previous findings, further research of nocebo treatment, exercise performance, and symptomatic responses following resistance exercise are warranted.

While the nocebo effect is largely attributed to psychological effects, physiological underpinnings may mediate responses to exercise. Elsenbruch et al. reported that negative expectations prior to a medical procedure reported higher anticipatory mental anxiety [8]. Through the induction of anxiety, neural activation may be altered in parts of the brain important in motor control and movement [14,15]. Indeed, negative expectations associated with the nocebo effect have been shown to induce anxiety, thereby increasing cholecystokinin (CCK) release [16]. Increases in CCK may lead to hyperalgesia and decrease extinction learning [17]. In the context of sport, hyperalgesia may lead to higher perceived exertion by athletes [18]. Decreases in extinction learning, or the ability to attenuate responses to negative stimuli, could hinder acclimation to novel high-intensity training programs [19]. Other physiologic explanations of the nocebo effect have suggested changes in endogenous opioid and dopaminergic responses, but how this influences exercise performance is unknown [20].

Negative expectations have been established to cause hyperalgesia due to underlying psychological and physiological mechanisms [4,21]. Exercise capacity may also be diminished due to the anticipation of negative outcomes associated with the nocebo effect [7,11,12,22]. Unaccustomed exercise has been shown to cause muscle soreness and decrease subsequent exercise performance [23]. However, it is currently unknown whether negative expectations following unaccustomed resistance exercise exacerbate adverse symptoms and whether possible changes manifest themselves in physical or psychological changes. Given that nocebo effects have been proposed to negatively influence muscle pain associated with statin use [9], detrimental expectations of post-exercise muscle soreness may decrease pain tolerance and exercise capacity following atypical resistance exercise. Thus, the purpose of this pilot study was to investigate nocebo effects on perceived muscle soreness, range of motion (ROM), RPE, and exercise capacity 48 h after unaccustomed resistance exercise. Furthermore, it was hypothesized that the negative expectations would increase muscle soreness, decrease ROM, decrease total repetitions, and increase RPE 48 h post-exercise.

## 2. Materials and Methods

### 2.1. Study Design

Untrained males (*N* = 14) were randomly assigned into one of two groups via simple random sampling: (1) negative-belief (*n* = 7), (2) control (*n* = 7) in a between-groups study design. Participants completed two trials each separated by 48 h apart. On the first trial, participants completed a supplement belief questionnaire, baseline perceived soreness measurement, and baseline range of motion (ROM) recording. Following this, an eccentric bicep curl pyramid was completed, and total repetitions and rate of perceived exertion (RPE) were recorded. After 48 h elapsed, participants repeated all methods sans questionnaire. At the end of the second trial, participants in the negative-belief group completed a questionnaire on the efficacy of the negative-belief treatment. Mean outcome values were compared between treatments and time points.

### 2.2. Participants

Untrained males (age = 20.5 years ± 0.9, height = 177.5 cm ± 5.5, body mass = 79.5 kg ± 11.9) from the surrounding area were recruited via convenience sample to participate in this study. Untrained was defined as completing ≤1 day of resistance training per week [24]. Suitability of exercise was determined using a modified physical activity questionnaire (PARQ) which excluded those reporting an upper extremity injury in the past 6 months, metabolic disease, musculoskeletal disease, cardiovascular disease, or any health problem which would affect muscle soreness or exercise capacity. Prior to each visit, participants were asked to refrain from caffeine, nicotine, and alcohol 12 h prior [25]. Prior to any data collection, verbal and written informed consent was obtained from each participant. All experimental procedures were conducted in accordance with the Declaration of Helsinki and approved by the Samford University Institutional Review Board (IRB) (EXPD-HP-19-SUM-8; May 23th 2019).

### 2.3. Negative-Belief and Control Treatment

For the negative-belief group, participants ingested a size “00” clear capsule filled with 400 mg of a white inert substance (gluten-free cornstarch) before the commencement of the exercise. Participants were told that the inert substance was an “inorganic nitrate” which would increase blood flow and inflammation following exercise leading to increased muscle soreness and decreased exercise performance for their follow-up visit 48 h later [12]. This would enable the nocebo effect. Treatment was administered by the same researchers for all participants. At the end of their second visit, participants in the negative-belief group were asked to answer two separate 5-point Likert scale questions regarding their perception of the inorganic nitrate (negative-belief) efficacy: (1) The inorganic nitrate made my exercise performance suffer, (2) The inorganic nitrate made my muscle soreness worse. For the control group, participants were only informed of the procedures and were not given any experimental treatment. Exercise protocols and procedures were identical between groups aside from treatment.

### 2.4. Procedures

For the initial visit, participant’s age, height, and weight were recorded. All participants answered two 5-point Likert scale questions regarding supplement beliefs: (1) I believe that using nutritional supplements can affect exercise performance, (2) I believe that using nutritional supplements can affect muscle soreness. Baselines soreness of the biceps brachii was assessed using an algometer and pain scale. Briefly, a bolster was placed behind the elbow, and an algometer (Wagner instrument, Greenwich, CT, USA) was pressed (40 N) into the mid-point of the muscle belly of the non-dominant arm at relaxed, full extension. On a scale of 1 (no pain) to 10 (worst pain possible), participants rated perceived pain on a visual analog scale. Baseline extension and flexion were measured using a goniometer (Elite Medical Instruments Inc., Anaheim, CA, USA). The goniometer was aligned proximally with the head of the humerus and distally with the radial styloid [26]. Participants were instructed to relax their arms completely and the maximum joint angle was determined at the point of subjective discomfort during elbow flexion and extension [27]. Passive range of motion was calculated using the difference of the angle of maximum extension minus the angle of maximum flexion. Following all baseline measures, participants completed an eccentric bicep curl pyramid previously described by Allen et al. [26]. Starting at full elbow flexion, participants lowered a 13.5 kg (30 lb) dumbbell with their non-dominant arm over 3 s to the pace of a metronome at 60 bpm. After a full elbow extension was reached, the researchers assisted the participant to return the dumbbell to the beginning position. Participants completed as many repetitions as possible until they could no longer keep pace with the metronome. This repeated while descending 2.25 kg (5 lbs) each set until a total load of 2.25 kg (5 lb) was reached. At the 2.25 kg total load, participants completed as many repetitions to fatigue or until a total of 10 repetitions were completed. Total repetitions and rate of perceived exertion (RPE; 1–10 scale) were documented at the end of every set and aggregated for analysis. Following the exercise protocol, participants were given an instructional care sheet directing them to avoid the following: Taking an oral anti-inflammatory (ibuprofen, acetaminophen, naproxen sodium), taking any “natural” pain relievers such as Vitamin C or ginger, applying any topical analgesic or pain relief cream, applying any ice or heat to the sore area, and massaging or intentionally stretching the sore muscle [28]. All participants completed a second visit 48 h after the initial visit where all procedures were repeated, and outcomes measured identically.

### 2.5. Statistical Analysis

All data were analyzed using Jamovi software (Version 0.9). A 2 × 2 (Treatment × Time). Repeated-measures ANOVA was used to statistically analyze all data with a Tukey post-hoc analysis as needed. Estimates of effect size for main effects were calculated using eta squared (η^2^). If warranted, Cohen’s d effect sizes (ES) were calculated between conditions and interpreted as: 0.2–0.49—small; 0.5–0.79—moderate; ≥0.8—large [29,30]. Significance was set at *p* ≤ 0.05 a priori. All data were presented as mean ± standard deviation (SD).

## 3. Results

For the supplementation belief questions, participants in the negative-belief group agreed that supplementation could influence exercise performance more than the control group (control = 4.0 ± 0.5, Nocebo = 4.6 ± 0.5; *p* = 0.045; ES = 1.12). Furthermore, participants in the negative-belief group agreed that supplementation could affect muscle soreness more than the control (control = 4.0 ± 0.5, Nocebo = 4.7 ± 0.7; *p* = 0.044; ES = 1.15). For the beliefs of the efficacy of the negative-belief associated with treatment (*n* = 7), 43% of participants agreed, and 57% strongly agreed that the “inorganic nitrate” made their exercise performance worse. No participants answered neutral, disagree, or strongly disagree. Additionally, 14% of participants agreed, and 86% strongly agreed that the “inorganic nitrate” made their muscle soreness worse. No participants answered neutral, disagree, or strongly disagree.

**Figure 1:**
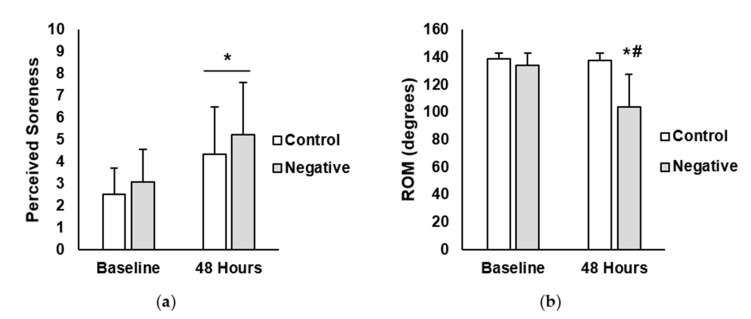
(**a**) Perceived muscle soreness in the control and negative-belief (negative) groups at baseline and 48 h after the soreness exercise protocol. (**b**) Range of motion (ROM) in control and negative-belief (negative) groups at baseline and 48 h after the soreness exercise protocol. Data are presented as mean ± SD. * indicates significantly different from baseline, # indicates significantly different from control (*p* < 0.05).

There were no significant differences at baseline for any of the variables. Perceived muscle soreness and range of motion (ROM) outcomes are presented in Figure 1. For perceive muscle soreness, there was a main effect for time (*p* < 0.001; η^2^ = 0.23). No significant main effects were seen for treatment (*p* = 0.462; η^2^ = 0.32) or interaction between treatment and time (*p* = 0.730; η^2^<0.01). For ROM (degrees), there was a main effect for time (*p* = 0.007; η^2^ = 0.11) and treatment (*p* = 0.037; η^2^ = 0.20). Furthermore, there was a significant interaction between time and treatment (*p* = 0.010; η^2^ = 0.10). Post-hoc analysis revealed that ROM in the negative-belief group was significantly lower at the 48-h time point compared to negative-belief baseline (negative-belief baseline = 133.9 degrees ± 9.2, negative-belief 48 h = 104.0 degrees ± 23.4; *p* = 0.004; ES = 1.83). No differences existed between time points for the control group (control baseline = 138.5 degrees ± 4.3, control 48 h = 137.7 degrees ± 5.4; *p* = 0.999; ES = 0.16).

**Figure 2:**
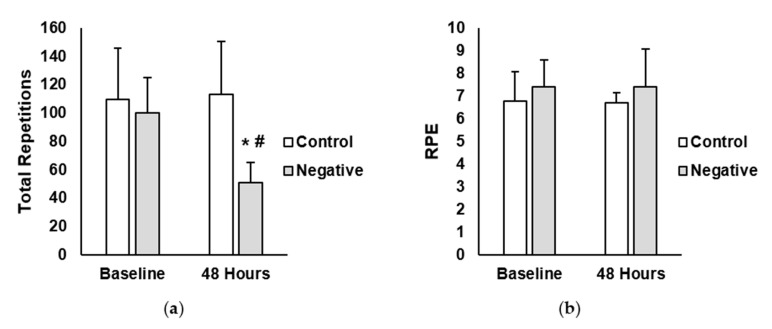
(**a**) Total repetitions completed in the control and negative-belief (negative) groups at baseline and 48 h after the soreness exercise protocol (**b**) Average rate of perceived exertion (RPE) in the control and negative-belief (negative) groups at baseline and 48 h after the soreness exercise protocol. Data are presented as mean ± SD. * indicates significantly different from baseline, # indicates significantly different from control (*p* < 0.05).

Total repetitions and rate of perceived exertion (RPE) are shown in Figure 2. A main effect for time (*p* < 0.001; η^2^ = 0.09) and treatment (*p* = 0.041; η^2^ = 0.28) was observed for total repetitions (reps). Additionally, there was a significant interaction between time and treatment for total repetitions (*p* < 0.001; η^2^ = 0.08). Post-hoc analysis showed that total repetitions in the negative-belief group was significantly lower at the 48 h time point compared to the negative-belief baseline (negative-belief baseline = 100 reps ± 25, negative-belief 48 h = 51 reps ± 14; *p* < 0.001; ES = 2.51). No differences existed between time points for the control group (control baseline = 110 reps ± 36, control 48 h = 113 reps ± 37; *p* = 0.999; ES = 0.08). For RPE, there were no main effects for time (*p* = 0.891; η^2^ < 0.01) or treatment (*p* = 0.282; η^2^ = 0.07). Moreover, no interaction between time and treatment were found (*p* = 0.890; η^2^ < 0.01).

## 4. Discussion

The nocebo effect has been well described to cause hyperalgesia [2]. Effects can decrease pain tolerance locally or more systemically [8,10]. Recent evidence has suggested that the nocebo effect is involved in increasing subjective muscle symptoms associated with statin use [9,31]. Furthermore, negative-belief and the nocebo effect has been shown to adversely impact athletic performance in various sports and modes of exercise [12,22]. It is currently unknown how negative-belief influences perceived soreness and exercise performance following unfamiliar resistance exercise. Thus, the purpose of this study was to gain insight into the effects of negative-belief on perceived soreness, range of motion (ROM), rate of perceived exertion (RPE), and exercise performance 48 h after unaccustomed resistance exercise. Findings reveal that negative-belief decreased ROM and total repetitions performed 48 h after unfamiliar resistance exercise. However, negative-belief did not change RPE during exercise or worsen muscle soreness 48 h beyond that of the controls. Distinct physiological mechanisms are not known at this time, but these findings have important implications for psychosomatic influences of responses to post-exercise induced muscle soreness.

The eccentric bicep curl pyramid protocol increased muscle soreness 48 h post-exercise. However, these changes were independent of treatment, suggesting that both the control and negative-belief groups perceived soreness similarly. This confirms previous findings that the current exercise protocol causes increased soreness 48 h later [26]. Counter to our hypothesis; the negative-belief did not worsen soreness compared to the controls. A possible explanation may be the extinction of the nocebo effect due to lack of reinforcement throughout the 48-h period between sessions. Although time course may differ based on the category of stimuli, previous evidence has shown that psychological response expectancies may be time-dependent [32]. Furthermore, Colagiuri et al. showed that hyperalgesia in response to nocebo treatment during electrodermal stimulation is most pronounced with continuous reinforcement rather than partial [33]. Thus, the time elapsed between visits and lack of reinforcement may have led to a less pronounced nocebo effect. However, it should be acknowledged that previous studies investigating the extinction of placebo/nocebo effects had numerous repeat visits leaving the contribution of nocebo extinction on perceived muscle soreness following exercise for a single visit unknown. Additionally, evidence has shown that previous experience or conditioning to painful stimuli may influence the efficacy of negative-beliefs [34,35,36]. Reicherts et al. showed that sensory pain ratings were only increased with negative-belief if the participants were conditioned to a thermal pain stimulus in addition to having negative expectations [35]. Since the current participants were sedentary, they may not have been conditioned to the muscle soreness pain, thereby dampening the nocebo effect. However, participant’s past training status or degree of experience with post-exercise muscle soreness of elbow flexors was not measured, leaving the contribution of conditioning unknown. Future investigations should seek to determine if prior experience with the stimuli which is used to induce the nocebo effect influences the response after novel resistance exercise.

Despite no treatment differences for muscle soreness, ROM was impaired in the negative-belief group. This is in accordance with previous findings that negative muscle and joint symptoms may be exacerbated through nocebo effects [9,37]. Manchilanti et al. showed that negative-belief of vertebral joint pain decreased the ability to perform movement before patients felt the sensation of pain [37]. While mechanisms for changes in ROM were not explicated in the present study design, anxiety has been reported to contribute to detrimental joint and muscle symptoms and treatment improving joint range of motion may accompany decreases in anxiety [38,39]. A primary mechanism of the nocebo effect is verbal-induced anxiety for perceived negative outcomes causing the release of cholecystokinin (CCK), thereby accentuating pain and sensation responses [2]. Furthermore, previous functional magnetic resonance imaging (fMRI) evidence has revealed that regions of the brain important in sensing somatosensory information (i.e., somatosensory cortex, insular cortex) exhibit hyperactivity under negative expectation conditions [14]. Since negative expectations may increase anxiety and intensify somatosensory information, thereby exacerbating the nocebo effect [4], changes in ROM could in part be due to increased joint symptoms although further investigation is needed to be conclusive.

Total repetitions 48 h after unaccustomed resistance exercise was lower with the negative-belief versus the control. This is in agreement with previous findings showing that negative expectations with nocebo conditions decrease exercise performance in athletes and physically active males [12,40]. Pollo et al. reported that sham electrical stimulation of the quadriceps significantly decreased total work during knee extension exercise [40]. Important to the current investigation, these findings are like the present in that isolated single-joint exercises were used, and differences in performance were found with negative expectation only, rather than expectation and conditioning. However, previous investigations have suggested that psychological determinants of exercise performance may be dependent on the intensity and muscle mass used [41,42]. More research is needed to determine if detrimental nocebo effects apply to other exercise modes and the amount of muscle involvement in movements. Although speculative, previous investigations have suggested that changes in performance with negative-belief may be supported by the “central governor” (CG) theory of control of movement whereby the CG controls physical limit by stimulating or inhibiting muscle activity [40,43]. Given that negative expectations have been shown to alter neural activation through conscious and unconscious mechanisms [44], negative-belief may have limited muscle activation through neural modulation. Albeit in a different clinical population, nocebo-induced bradykinesia has been documented [2]. Nocebo effects on alteration of muscle activity should be the aim of subsequent investigations.

In the current investigation, RPE was unaltered regardless of treatment. This supports others investigating nocebo effects on physical performance [40]. While Bottoms et al. found local RPE of active muscle was higher with nocebo treatment, no differences were found for central RPE, which reflects the overall exertion of the body [5]. Our results reinforce this in that no differences in central RPE were noted currently. It is plausible that central RPE may not be a limiting mechanism with negative expectations, while local RPE may be a larger factor of performance. Indeed, anxiety may be a key mechanism for nocebo effects, and previous studies have shown that lower anxiety scores may decrease local intensity ratings during cycle ergometry [45]. Thus, a more in-depth study of negative-belief and local/central RPE is warranted. Interestingly, all participants in the negative-belief group agreed or strongly agreed that the “supplement” negatively impacted soreness and performance. Furthermore, the negative-belief group agreed that supplements could influence soreness and performance more than the control group. This reinforces Hurst et al., and their findings in that placebo/nocebo efficacy is determined by the individuals intent and beliefs of supplement use [12]. This may be in part due to individual experiences or conditioning with supplement use in the past. Previous experience with a drug has been shown to create memories of effective or ineffective treatments [46]. Participants in the negative-belief group may have had more experiences with supplements changing performance, thus affecting the potency of the nocebo effect. Intent to use or current use of supplementation was not directly measured in the current investigation and should be controlled for in subsequent studies.

While the current study presents novel data concerning nocebo effects and post-exercise muscle soreness responses following unaccustomed physical activity, there were some limitations. Most notably, the sample size in the current investigation was limited, which may not be generalizable to larger populations. However, these results show novel subjective and objective data on the nocebo effect and resistance exercise, which may serve to direct future large scale investigations. A physiological mechanism for changes due to nocebo treatment was not determined using the present study design. Although physiological mechanisms for nocebo effects have been well studied [4,16,47], there are several possible factors which could have led to changes leaving the contribution of the various mechanisms unknown. Thus, previously discussed physiological mechanisms remain largely speculative necessitating deeper study. Moreover, only one specific joint and mode of exercise was studied. Previous reports have shown differences in nocebo efficacy with other exercise modes [5]. Thus, results may not be generalizable to other modes of exercise with multi-joint movements. Supplement beliefs were different between groups, and this is supported by other previous literature investigating nocebo effects on exercise [12]. However, due to sample size, covariate analysis was not possible in the current investigation, leaving the need for more investigation related explicitly to how supplement beliefs influence nocebo effects during resistance exercise.

## 5. Conclusions

In conclusion, the current pilot study presents new information on how the nocebo effect contributes to changes in exercise response following unaccustomed activity. Nocebo effects in sport and exercise, especially resistance exercise, are unclear, and these early findings suggest that negative expectations may induce adverse responses following unaccustomed activity. Specifically, findings reveal that negative-belief adversely impacts performance and ROM following unfamiliar resistance exercise while RPE and soreness were not exacerbated beyond controls. From a practical standpoint, these data may hold important implications for coaches and practitioners. Based on current findings, coaches and practitioners should avoid inducing negative expectations during training. For example, if coaches emphasize to their athletes that a training session will be “extremely hard” or “make them sore”, it is possible that this may have an impact on performance even days later. However, more follow up study using larger sample sizes on the nocebo effect, and resistance exercise is needed specifically on what induces negative expectations and how this may affect training responses in the field. These findings may serve as a launchpad for new research on psychological influences on resistance exercise performance.
